# Identification of GSK3β inhibitor kenpaullone as a temozolomide enhancer against glioblastoma

**DOI:** 10.1038/s41598-019-46454-8

**Published:** 2019-07-11

**Authors:** Tomohiro Kitabayashi, Yu Dong, Takuya Furuta, Hemragul Sabit, Shabierjiang Jiapaer, Jiakang Zhang, Guangtao Zhang, Yasuhiko Hayashi, Masahiko Kobayashi, Takahiro Domoto, Toshinari Minamoto, Atsushi Hirao, Mitsutoshi Nakada

**Affiliations:** 1 Department of Neurosurgery, Faculty of Medicine, Institute of Medical, Pharmaceutical and Health Sciences, Kanazawa University, Kanazawa, Japan; 2 Department of Pathology, Kurume University School of Medicine, Kurume, Japan; 3 Division of Molecular Genetics, Cancer Research Institute, Kanazawa University, Kanazawa, Japan; 4 Division of Translational and Clinical Oncology, Cancer Research Institute, Kanazawa University, Kanazawa, Japan; 5 Department of Neurosurgery, The First Affiliated Hospital of Harbin Medical University, Harbin, People’s Republic of China; 6 Department of Neurosurgery, The First Hospital of Jilin University, Changchun, People’s Republic of China

**Keywords:** Drug development, CNS cancer

## Abstract

Cancer stem cells are associated with chemoresistance and rapid recurrence of malignant tumors, including glioblastoma (GBM). Although temozolomide (TMZ) is the most effective drug treatment for GBM, GBM cells acquire resistance and become refractory to TMZ during treatment. Therefore, glioma stem cell (GSC)-targeted therapy and TMZ-enhancing therapy may be effective approaches to improve GBM prognosis. Many drugs that suppress the signaling pathways that maintain GSC or enhance the effects of TMZ have been reported. However, there are no established therapies beyond TMZ treatment currently in use. In this study, we screened drug libraries composed of 1,301 existing drugs using cell viability assays to evaluate effects on GSCs, which led to selection of kenpaullone, a kinase inhibitor, as a TMZ enhancer targeting GSCs. Kenpaullone efficiently suppressed activity of glycogen synthase kinase (GSK) 3β. Combination therapy with kenpaullone and TMZ suppressed stem cell phenotype and viability of both GSCs and glioma cell lines. Combination therapy in mouse models significantly prolonged survival time compared with TMZ monotherapy. Taken together, kenpaullone is a promising drug for treatment of GBM by targeting GSCs and overcoming chemoresistance to TMZ.

## Introduction

Glioblastoma (GBM) is the most common and most malignant adult primary brain tumor. Despite development of surgical techniques and several anti-cancer drugs, GBM prognosis is still poor. Temozolomide (TMZ), an alkylating agent, markedly improved survival time, as demonstrated by Stupp *et al*.^1^. Thus, TMZ has been used as a standard chemotherapy since 2005. However, GBM cells acquire resistance and become refractory to TMZ ultimately during treatment. Overall survival (OS) of GBM patients is less than 2 years in most cases^2,3^. One of the hypothesized causes of drug resistance and short interval of recurrence of several cancer types is the existence of cancer stem cells. These types of stem cells are present in GBM, and are termed glioma-initiating cells or glioma stem cells (GSCs)^4^. Because GSCs are tumorigenic, highly invasive, and maintain the tumor, GBM shows high resistance to chemo-radiotherapy. Thus, GSCs are a promising therapeutic target with potential to completely cure GBM^5^. However, although targeting GSCs provides benefits *in vitro*^6^, *in vivo* evidence of targeting GSCs in clinical use has not been reported. Therefore, it is important to establish new therapies targeting GSCs. As there are no drugs beyond TMZ for GBM, development of strategies for enhancing the effect of TMZ to overcome drug resistance and improve survival time is essential.

Drug development is costly and success is not guaranteed. To reduce cost and improve success rates, the concept of “drug repositioning” or “drug repurposing” has gained attention as an approach to drug discovery. This concept refers to identification of new indications for already approved drugs and offers the benefits reducing costs and decreasing time required to get the drug approved^7^. To discover compounds efficiently, we established a screening system for drugs that target cancer stem cells using existing drug libraries^8,9^.

In this study, we screened existing drugs that enhance the effects of TMZ on GSCs with the goal of drug repositioning. We identified the drug kenpaullone, an inhibitor of glycogen synthase kinase (GSK) 3β^10,11^.

## Results

### Potential candidate compounds that enhance effects of TMZ against GSCs

**Figure 1:**
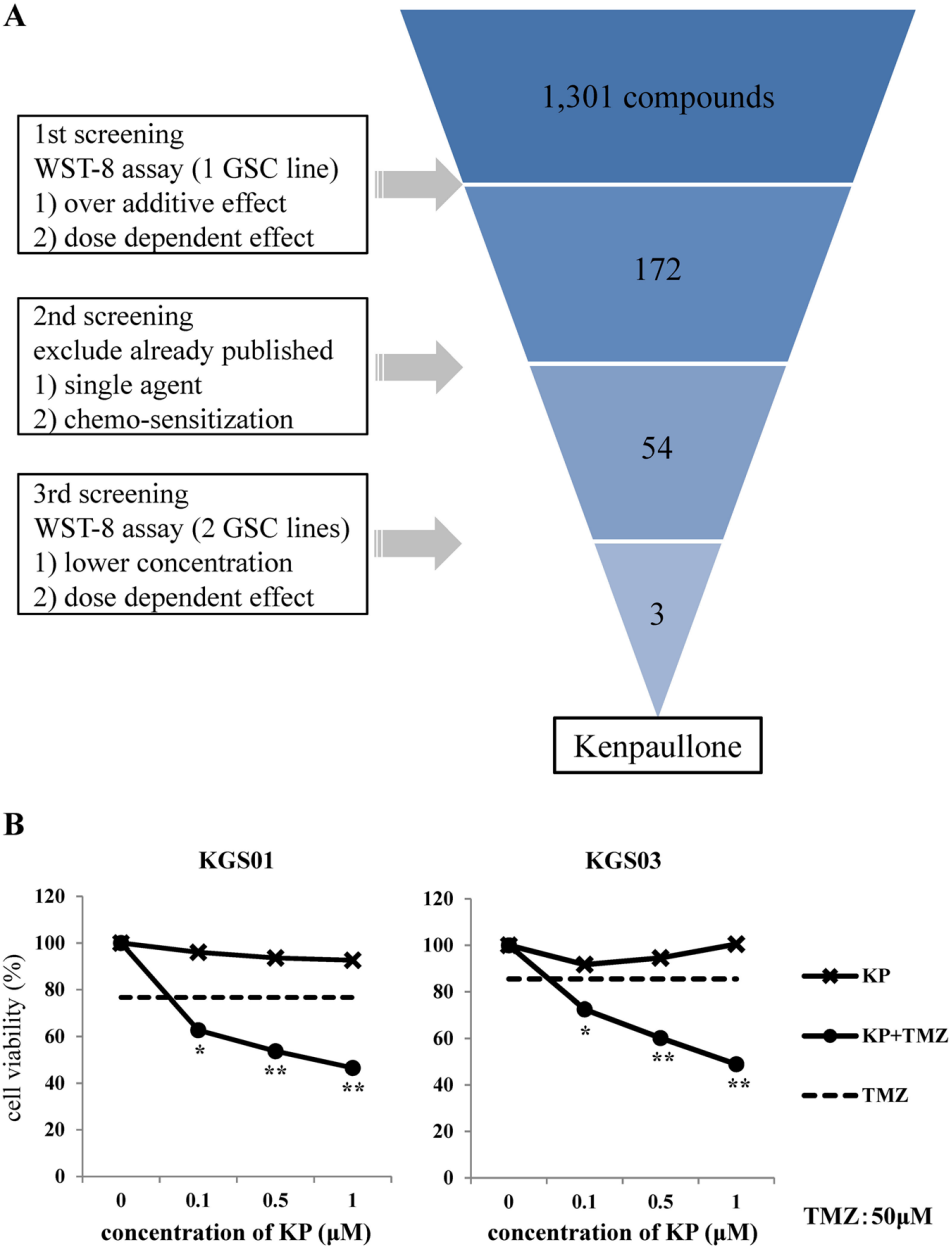
Schematic representation of the drug screening procedure and its results. A total of 1,301 compounds from five libraries were screened in three steps. The results of screening of kenpaullone in two GSCs are presented in the lower panels. The dotted line shows effect of TMZ (50 μM). *p < 0.05, **p < 0.01. KP: kenpaullone.

We performed cell viability assays to screen drugs identified in existing libraries. Of the 1,301 compounds identified, 172 showed various degrees of TMZ-enhancing effects against viability of GSCs as estimated by the 2-(2-methoxy-4-nitrophenyl)-3-(4-nitrophenyl)-5-(2,4-disulfophenyl)-2H-tetrazolium (WST-8) assay. In the second step of screening, we excluded drugs that have been previously reported to show effects alone or in combination with TMZ against GBM, resulting in 54 remaining candidate compounds. Finally, three drugs that exhibited strong effects for enhancing TMZ at lower concentrations remained (Fig. 1A). From these three compounds, kenpaullone (9-bromo-7,12-dihydroindolo[3,2-d][1]benzazepin-6(5H)-one) was selected because of its novelty. Additionally, since molecular weight of kenpaullone is low (327.18 g/mol) and kenpaullone might be highly lipophilic because of insolubility in water or ethanol and its structural formula containing two benzene rings, kenpaullone was inferred to pass through the blood brain barrier (BBB). The other two candidate compounds are being investigated in other studies.

WST-8 assay showed that combination therapy with kenpaullone and TMZ decreased the viability of both GSC lines in a dose-dependent manner (Fig. 1B). Although TMZ and kenpaullone each alone reduced cell viability by approximately 10%, combination therapy reduced cell viability by 30–50% (*p* < 0.01).

**Figure 2:**
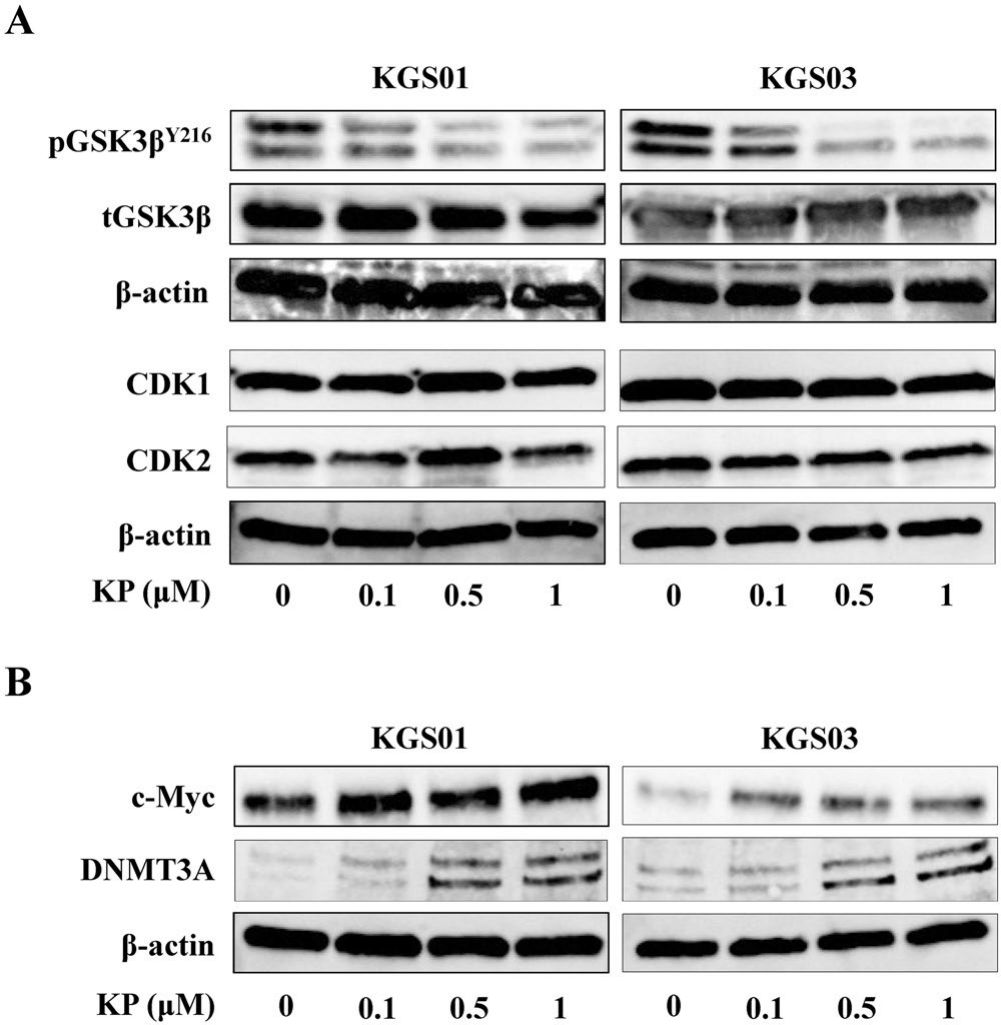
Confirmation of the effects of kenpaullone as a kinase inhibitor. (**A**) Kenpaullone inhibited GSK3β but did not inhibit CDK 1/2 at the indicated concentrations. Expression of β-actin was monitored as a loading control. Most of the Bands were cropped from different parts of the same gel, and few from different gels. CDK1/2: cyclin-dependent kinase 1/2, GSK3β: glycogen synthase kinase 3β, KP: kenpaullone. (**B**) Levels of c-Myc and DNMT3A in GSCs were increased by kenpaullone. Bands were cropped from different parts of the same gel, or from different gels. DNMT3A: DNA methyltransferase 3A, KP: kenpaullone.

Kenpaullone is a GSK3β and cyclin-dependent kinase (CDK) inhibitor^10–13^. Therefore, we examined these inhibitory effects of kenpaullone on GSC cell lines at first after screening. GSK3β is activated by phosphorylation of tyrosine 216 (Y216) residue (pGSK3β^Y216^). Kenpaullone decreased the level of pGSK3β^Y216^ in a dose-dependent manner, but did not decrease expression of CDK1 and CDK2 in GSCs (Fig. 2A). We previously reported that GSK3β inhibition sensitizes human GBM cell lines to TMZ through up-regulation of DNA methyltransferase 3A (DNMT3A) that methylates O-6-methylguanine methyltransferase (MGMT) promoter via c-Myc up-regulation^14^. Kenpaullone induced expression of c-Myc and DNMT3A in both KGS01 and KGS03, as expected (Fig. 2B).

These results suggest that kenpaullone inhibits GSK3β in GSCs and has therapeutic potential against GSCs as a TMZ enhancer.

### Attenuation of stemness and viability of GSCs by kenpaullone

**Figure 3:**
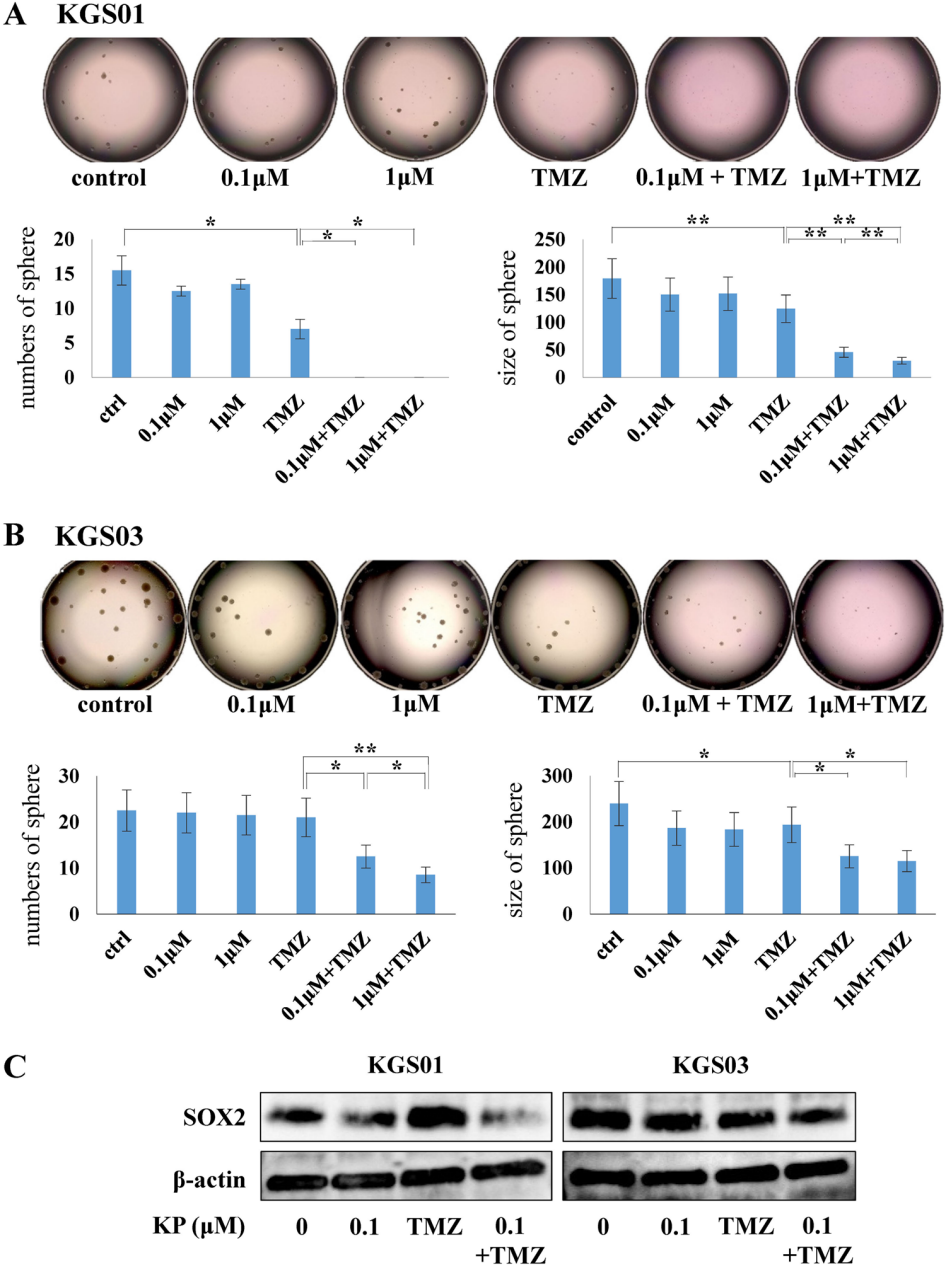
Kenpaullone suppressed sphere-forming ability of two GSC lines in combination with TMZ. (**A**) Tumor spheres of KGS01 treated with kenpaullone and/or TMZ for 10 days. Graphs show the number and size of spheres. (**B**) The results of KGS03 shown in the same way as KGS01. (**A,B**) Combination therapy markedly decreased sphere-forming ability of both GSCs, especially KGS01. *p < 0.05, **p < 0.01. (**C**) Western blotting analysis of the stem cell marker SOX2 in both GSCs. Combination therapy suppressed the expression of SOX2 more strongly than kenpaullone alone. β-actin was monitored as a loading control. Concentration of TMZ was 50 μM. Bands were cropped from different parts of the same gel, or from different gels. KP: kenpaullone.

Since kenpaullone showed inhibition of GSC viability, we analyzed its effects on stem cell properties. After 10 days treatment with kenpaullone, we estimated sphere-forming capacity of GSCs by measuring the number and size of spheres. For KGS01, both kenpaullone and TMZ inhibited sphere formation and TMZ showed stronger inhibition. Importantly, combination of kenpaullone and TMZ completely suppressed formation of spheres larger than 150 μm (Fig. 3A, *p* < 0.05). Sphere size was smaller in the TMZ group than the kenpaullone group (Fig. 3A, *p* < 0.01). For KGS03, neither number nor size of spheres was decreased by treatment with kenpaullone or TMZ alone but strongly decreased by combination treatment (Fig. 3B, *p* < 0.05). Interestingly, 0.1 μM kenpaullone significantly reduced both number and size of spheres in combination with TMZ in both GSCs, but not alone.

Previous studies have shown that SRY (sex determining region Y)-box (SOX) 2 is a key regulator of stemness in GSCs^15,16^. Western blotting demonstrated that combination treatment with TMZ and kenpaullone suppressed expression of SOX2, suggesting that reduced stem cell properties following combination therapy might be associated with suppression of SOX2 expression (Fig. 3C). These data indicated that kenpaullone, when combined with TMZ, suppressed the stem cell phenotype.

### TMZ-enhancing effect of kenpaullone on viability of GBM cells

**Figure 4:**
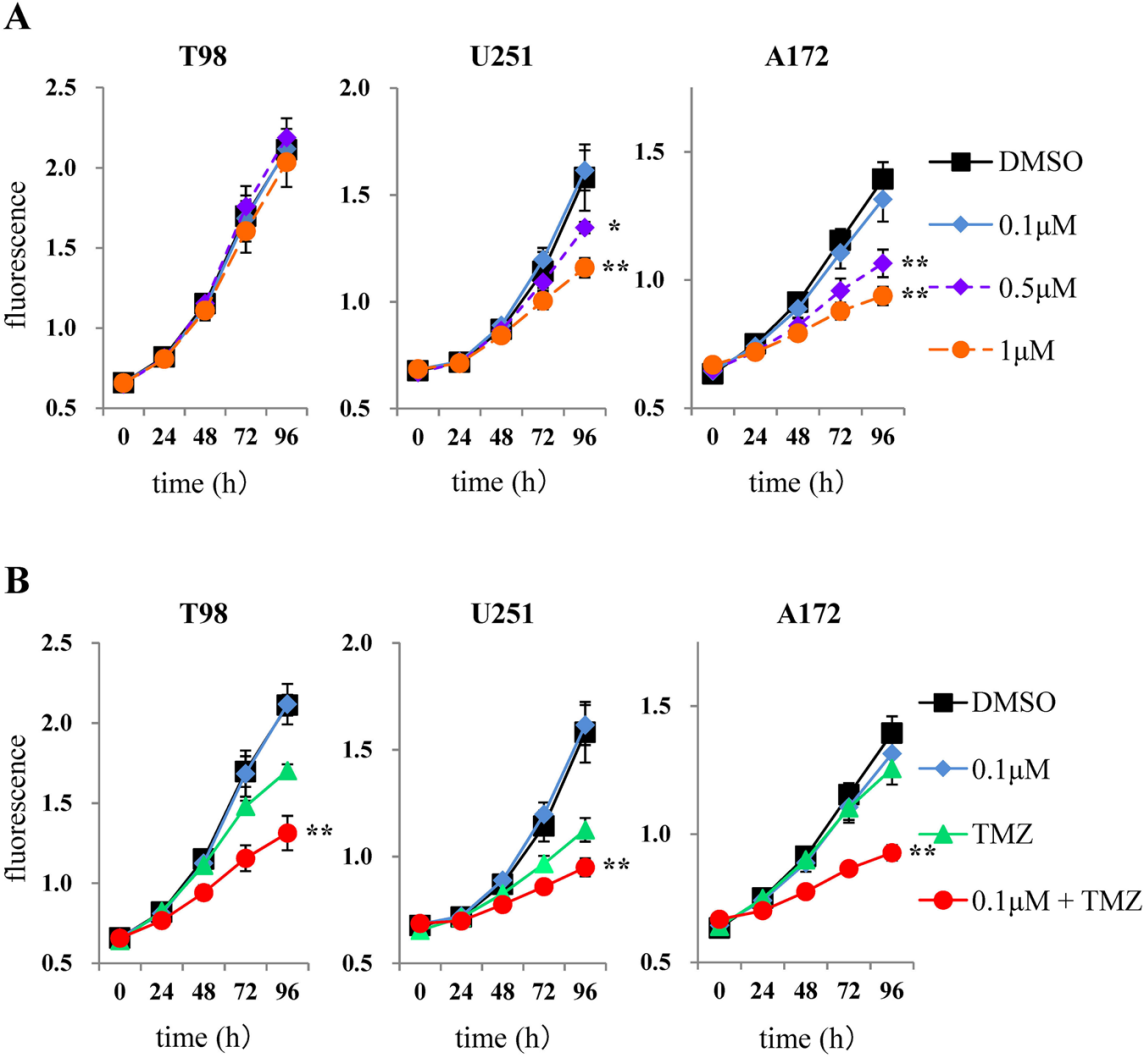
Effects of kenpaullone and enhancement of TMZ effect on viability of GBM cell lines. (**A**) Cells were treated with kenpaullone at 0.1, 0.5, and 1 μM and viability curves were analyzed at each concentration. (**B**) Cells were treated with a combination of kenpaullone (0.1 μM) and TMZ (50 μM). Kenpaullone potentiated TMZ effects on GBM cell viability. *p < 0.05, **p < 0.01.

When treating malignant glioma, the best strategy is to select drugs that inhibit not only GSCs, but also differentiated tumor cells because of interconversion between differentiated non-GSCs and GSCs in GBM^17^. Thus, we evaluated the efficacy of kenpaullone against GBM cell lines. Similar to GSCs, Y216 phosphorylation of GSK3β, but not expression of CDKs, was inhibited in all GBM cell lines by kenpaullone (Supplementary Fig. S2). Kenpaullone inhibited viability of U251 and A172 cells in a dose-dependent manner, but not T98 cells (Fig. 4A). When GBM cells were treated by TMZ in combination with 0.1 μM kenpaullone, a dose that did not inhibit viability with monotherapy, marked inhibition of viability was observed in all GBM cell lines (Fig. 4B). Taken together with the results using GSCs and GBM cell lines, low concentrations of kenpaullone have therapeutic potential for enhancement of TMZ effects against GBM, which are composed of GSCs and differentiated tumor cells.

### Apoptosis detection assay

**Figure 5:**
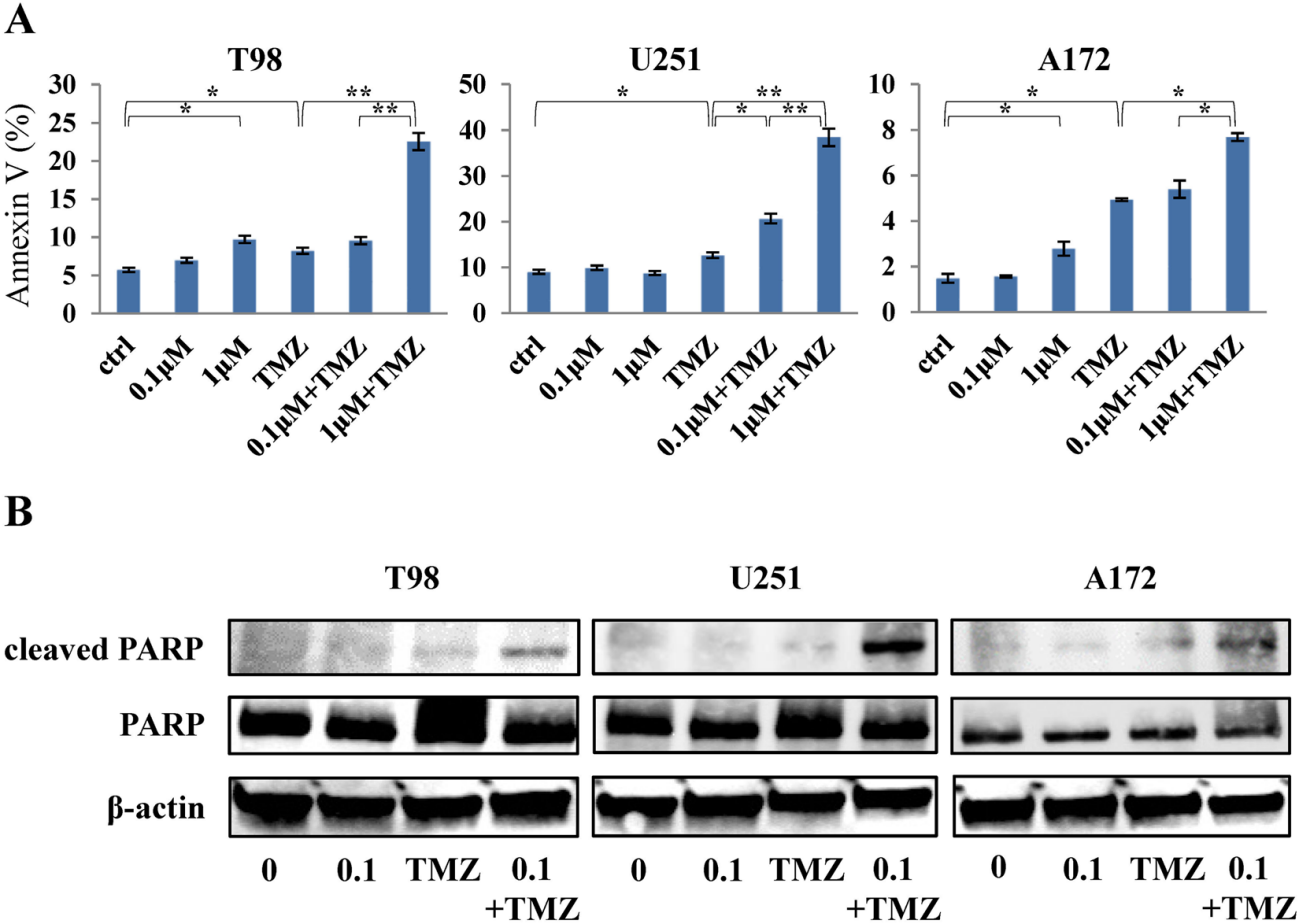
Combination therapy by kenpaullone and TMZ induced apoptosis. (**A**) Kenpaullone accelerated induction of apoptosis. Apoptotic cells were detected by flow cytometry in GBM cell lines following treatment with kenpaullone and/or TMZ for 48 h. The bar graphs show the ratio of apoptotic cells quantified by Annexin V. The vertical axis of the graph represents Annexin V-positive cells (%). ctrl; treatment with DMSO. *p < 0.05, **p < 0.01. (**B**) Expression levels of cleaved PARP in GBM cell lines were increased by combination therapy.

We assessed whether the reduction of viability by combination therapy with kenpaullone and TMZ was associated with induction of apoptosis since inhibition of GSK3β is related to apoptosis in tumor cells via several signaling pathways^18–21^. As expected, TMZ induced apoptosis and kenpaullone significantly enhanced the TMZ effect in a dose-dependent manner in all GBM cell lines, although kenpaullone itself showed only small effects on apoptosis induction (Fig. 5A). We confirmed by Western blotting that the expression level of apoptosis related molecule is similarly enhanced in combination group (Fig. 5B). This is consistent with the results of cell viability assays, suggesting that enhanced suppression of cell viability by kenpaullone might be attributed to induction of apoptosis in GBM cell lines.

### Mouse model treatment

**Figure 6:**
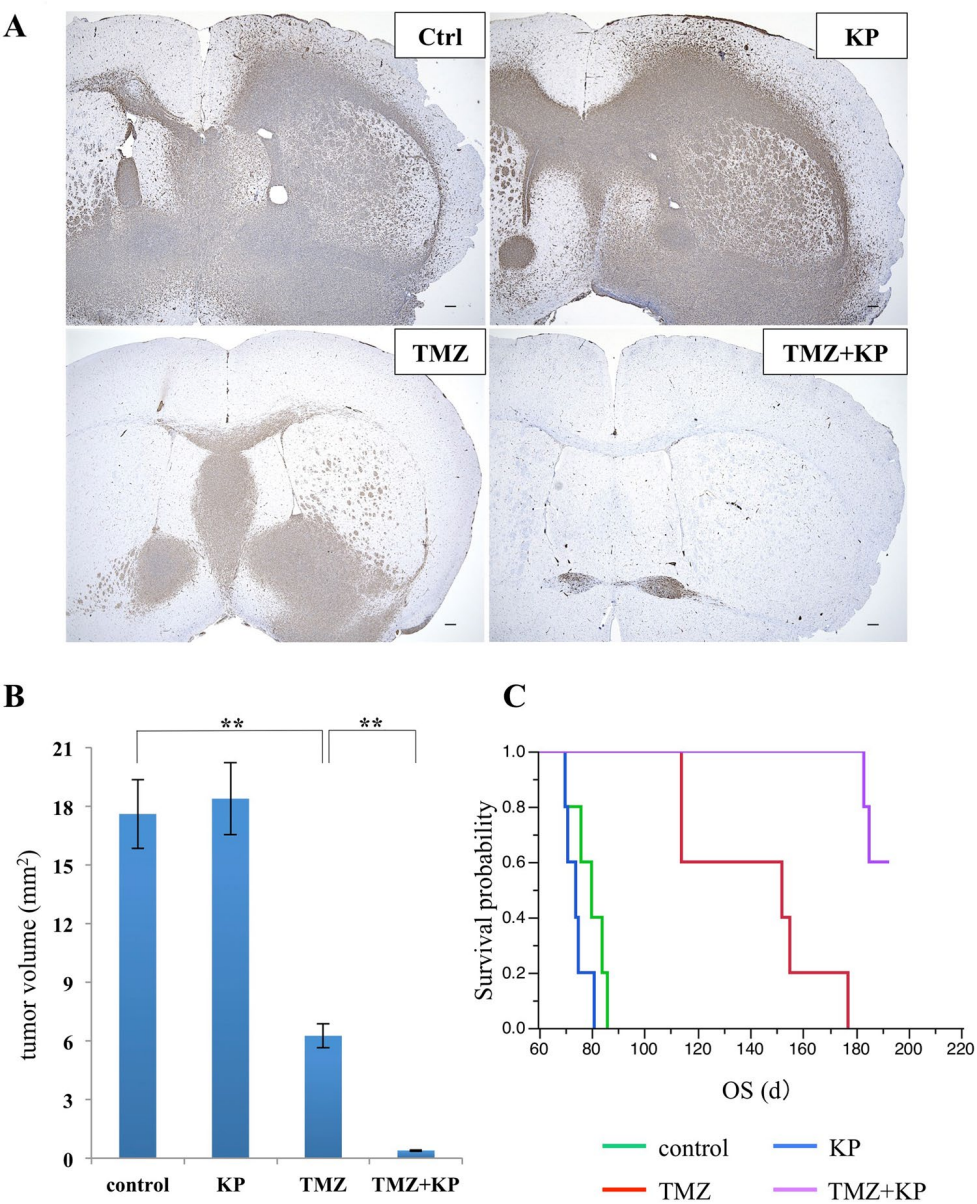
Effect of combination therapy by kenpaullone and TMZ in the GBM mouse model. (**A**) Representative histological sections of the brain tumors treated with kenpaullone and/or TMZ. Tumor cells were detected by immunostaining of nestin. Mice treated with combination therapy showed markedly reduced tumor volume. *Scale bar* = 100 μm. (**B**) Calculated maximal size of tumors was shown. Combination therapy resulted in a remarkable reduction of tumor size compared with the TMZ group. The vertical axis of the graph shows tumor area (mm^2^). **p < 0.01. (**C**) Survival time of mice treated with DMSO (control), kenpaullone, and/or TMZ. Combination therapy significantly prolonged survival of the KGS01 mouse model compared with control and monotherapy. Log-rank test, *p* < 0.0001. KP: kenpaullone.

To expand on our *in vitro* experiments, we evaluated the effects of kenpaullone *in vivo*. KGS01 was used for animal experiments because the TMZ enhancing effect was more prominent than KGS03 (Fig. 3). We conducted animal experiments in two steps. In the first step, we treated mice for 9 weeks, then, euthanized them and evaluated tumor formation. To identify diffusely infiltrating tumor cells, we immunostained specimens used antibody of nestin and measured tumor area (Fig. 6A). Tumor growth was suppressed in the TMZ group, but not in the kenpaullone group, compared to the control group. However, combination therapy significantly suppressed tumor growth by 97.8% compared to the control group (Fig. 6B, *p* < 0.01). In the second step, we performed the mouse model treatment again and continued treatment until mice died to evaluate survival time. TMZ significantly prolonged survival time compared to the control group (p = 0.0018). Even though the changes are not statistically significant and thought to be possible error, kenpaullone slightly decreased survival. Nevertheless, combination therapy significantly prolonged survival compared to the TMZ group (Fig. 6C, *p* = 0.0017). No obvious adverse events were observed in either *in vivo* study.

**Figure 7:**
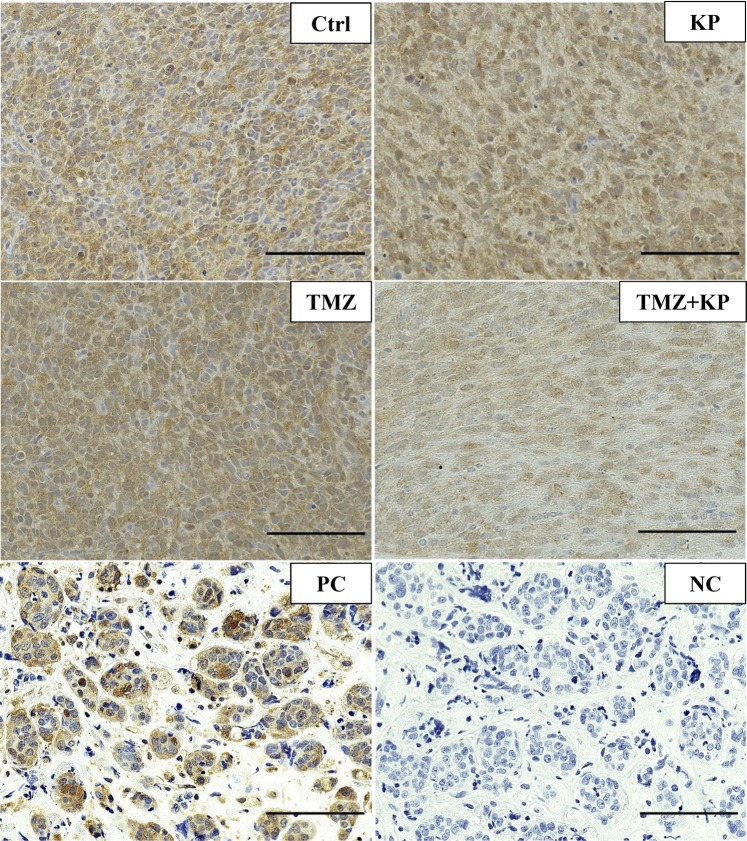
Immunohistochemistry for the active form of GSK3β in mouse brain tumor. The level of pGSK3β^Y216^ was not affected by kenpaullone or TMZ alone while the combination therapy markedly decreased pGSK3β^Y216^ in the tumor cells. Specimens of metastatic breast cancer were used as a positive control for immunostaining. *Scale bar* = 100 μm. Ctrl: tumor in a mouse treated with DMSO; KP: kenpaullone. PC: positive control and NC: negative control (non-immune rabbit IgG) for immunostaining of pGSK3β^Y216^.

To confirm the effect of kenpaullone *in vivo*, GSK3β level was analyzed using immunostaining of pGSK3β^Y216^ in tumor cells. Monotherapy with kenpaullone did not change the level of pGSK3β^Y216^. However, combination therapy decreased pGSK3β^Y216^ efficiently (Fig. 7). These results together with those of the therapeutic effect against the tumors (Fig. 6A,B) suggest that kenpaullone could get into the brain tumor with TMZ, and GSK3β inhibition might be the important mechanism for enhancing the effects of TMZ.

## Discussion

In this study, we used our screening system and selected kenpaullone, a GSK3β inhibitor, as a TMZ enhancer from drug libraries containing 1,301 compounds. Kenpaullone in combination with TMZ suppressed cell viability and sphere-forming ability of GSCs derived from patients, and their orthotopic tumor growth in mice. The combined therapy also efficiently inhibited cell viability via induction of apoptosis in GBM cell lines. Kenpaullone has been reported to have therapeutic potential for several cancer types such as breast and prostate cancers via inhibition of GSK3β and CDK^12,22–26^. However, no reports have demonstrated that kenpaullone has therapeutic potential against GBM or cancer stem cells. This is the first report that kenpaullone can be utilized as a TMZ enhancer via GSK3β inhibition for patients with GBM and as a good candidate for tumor stem cell targeting therapy.

Kenpaullone was developed as GSK3β and CDK inhibitor in 2003^27^. Although kenpaullone inhibited GSK3β in a dose-dependent manner in our study, CDK was not inhibited, in contrast to previous reports^10,12^. This might be attributed to the IC_50_ value, as the IC_50_ for inhibition of CDK by kenpaullone (0.4–0.8 μM) is much higher than that of GSK3β (0.023–0.08 μM)^10–13^. The concentration used in the present study might not be high enough to inhibit CDK. Another possibility is that kenpaullone exerts effects in a cell type-dependent manner.

In this study, we demonstrated that kenpaullone enhanced the ability of TMZ to attenuate GSCs property. We previously demonstrated that GSK3β inhibition enhances TMZ effects by silencing MGMT expression via c-Myc-mediated promoter methylation^14^. Kenpaullone showed similar effects, possibly through GSK3β inhibition, in GSCs. This may explain TMZ enhancement by kenpaullone. Another mechanism might be the downregulation of SOX2 as shown in Fig. 3C. Combination therapy decreased expression of SOX2, which is consistent with attenuation of stem cell properties. Previous reports demonstrated that GSK3β inhibitors suppress glioma stem cell properties via down-regulation of SOX2^28,29^. In this study, a slight decrease in SOX2 expression was observed with kenpaullone monotherapy, and TMZ did not inhibit SOX2 expression, suggesting that TMZ may inhibit stem cell phenotype by mechanisms other than regulation of SOX2. Analysis of the mechanism underlying the TMZ-enhancing effect by kenpaullone is ongoing in our laboratory.

Our study demonstrated that low dose of kenpaullone with TMZ effectively suppressed cell viability possibly due to induction of apoptosis in GBM cell lines. Previous reports showed that GSK3β inhibitors potentiate apoptosis via several mechanisms such as p53 induction^30^, inhibition of extracellular signal-regulated kinase (ERK)/Akt signaling, and c-jun N-terminal kinase (JNK) signaling^18,21,31^. TMZ induced c-Myc-mediated apoptosis via Akt signaling^32^. These results suggest that the TMZ-enhancing effect of kenpaullone on apoptosis may involve multiple signaling pathways.

In mouse model experiments, kenpaullone alone did not affect tumor growth and survival time. There are several possible reasons for these results. First, kenpaullone may not have reached the brain when administered alone. Drug must cross the sequential barriers formed by the BBB and blood-tumor barrier (BTB) to reach the tumor *in vivo*^33^. Our immunohistochemistry data showed no inhibition of GSK3β level by kenpaullone monotherapy (Fig. 7), which supports this hypothesis. Second, the concentration of kenpaullone may have been too low for monotherapy to inhibit GSK3β. The IC_50_ value for kenpaullone *in vivo* might be higher than *in vitro*^34,35^ or intraperitoneal injection of kenpaullone may require more volume to reach a high enough concentration to enter the brain. In contrast, kenpaullone seems to pass through the BBB when administered in combination with TMZ, as shown by immunostaining. This might be explained by TMZ-mediated down-regulation of P-glycoprotein in BBB cells by disrupting Wnt3 signaling, resulting in enhanced BBB permeability^36,37^.

Although the safety of kenpaullone for normal brain cells was not revealed, kenpaullone has been reported that has neuroprotective effect and therapeutic potential for neurodegenerative diseases^38–43^. Therefore, we believed that kenpaullone is at least not harmful to normal brain tissue.

As a limitation of this study, we only evaluated lower concentrations of kenpaullone compared to previous reports^23–26^. Determination of the optimal concentration and safety limit for GBM will be evaluated in future studies, since kenpaullone showed antitumor effects at higher concentrations in several cancer types^24,26^. Additional preclinical studies are needed to confirm that kenpaullone contributes to GBM treatment.

In conclusion, we identified kenpaullone, a GSK3β inhibitor, as a promising medicine to enhance the effects of TMZ against GSCs and differentiated GBM cells. GSK3β inhibition is a key factor in overcoming therapy resistance via multiple pathways^44^. Since our mouse model demonstrated successful results without adverse events, kenpaullone promises to be an effective and safe TMZ enhancer for GBM.

## Materials and Methods

### Cell culture

We used Dulbecco’s Modified Eagle’s Medium (DMEM) supplemented with 10% heat-inactivated fetal bovine serum (FBS) to culture A172, T98 and U251, and they were cultured in an incubator set to 37 °C with 5% CO_2_. These three human GBM cell lines were obtained from American Type Culture Collection (Manassas, WA, USA) and characterized at the Resource Institute by short tandem repeat profile analysis. Since these cell lines were expanded in fewer than two passages and stored frozen at −80 °C, authentication of them was unnecessary. We only used cells that have been passaged in small numbers for up to 6 months after thaw.

For GSCs, we established and used patient-derived GBM cell lines named KGS01 and KGS03. These cell lines were established at Kanazawa University by isolating from GBM specimens obtained during surgery from consenting patients. Isolation procedure was performed as same as previous reports^16,45,46^. In particular, obtained tissues were cut up and dissociated into single cells using Accutase. Secondary, the cells forming spheres in culture medium were picked up and the same procedure was repeated to establish a stem cell line. The project was approved by both human genome/genetic analysis research ethics committee and medical ethics committee of Kanazawa University (approval number: 209, 2080, 2188), and informed consent for participation in this study was obtained from each subject. All methods were performed in accordance with the relevant guidelines. We already have confirmed KGS01 as tumor-initiating cell line in previous study because of self-renewal ability *in vitro* and reproduction of the original tumor in a mouse xenograft model^9^. We confirmed KGS03 as GSCs with same procedure as KGS01. Specifically, we performed immunofluorescence assay for several markers. Antibodies used are shown in Supplementary Table 1. As a result, KGS03 formed spheres and expressed surface markers representing stemness, such as CD44, CD133, and SOX2 (Supplementary Fig. S1A)^4,15,16,47^. KGS03 can differentiate into both astrocyte-like cells and neuron-like cells when cultured in DMEM with 10% FBS (Supplementary Fig. S1A). These astrocyte-like cells presented glial fibrillary acidic protein (GFAP)- and oligodendrocyte transcription factor (Olig2)-positive, and neuron-like cells presented neuron-specific class III β-tubulin (Tuj1)-positive. Furthermore, KGS03 can grow into brain tumor cells with histological features of the original *in vivo* GBM (Supplementary Fig. S1B). Medium for neurosphere consists of DMEM/F12 (Gibco, Life Technologies, Carlsbad, CA, USA), recombinant human epidermal growth factor (EGF) at 20 ng/mL (Sigma–Aldrich, St. Louis, MO, USA), recombinant human basic fibroblast growth factor at 20 ng/mL (Sigma–Aldrich), B27 supplement without vitamin A (Gibco), and GlutaMAX (Gibco). GSCs were cultured in neurosphere medium using ultra-low attachment surface plates (Corning, Cambridge, MA, USA).

### Drug screening

We performed three steps screening of the following drug libraries against GSCs to detect potentially useful compounds (Fig. 1A): FDA (U.S. Food and Drug Administration)-approved drug library (640 compounds; CB-BML-2841J0100), ICCB (Harvard Institute of Chemistry and Cell Biology) known bioactives library (480 compounds; CB-BML-2840J0100), kinase inhibitor library (80 compounds; CB-BML-2832J0100), fatty acid library (68 compounds; CB-BML-2803J0100), and phosphatase inhibitor library (33 compounds; CB-BML-2834J0100). First, we treated one GSC line (KGS01) with each drugs at higher concentrations (1, 5, and 20 µM) with or without TMZ (Sigma–Aldrich) in a 384-well Corning ultra-low attachment plate (Corning) for 48 hours (h), and the rate of viable cells compared with control group were assessed using a WST-8 assay described in detail below. The compounds that demonstrated greater cell killing effect than additive effect when combined with TMZ were picked up. Second, we excluded drugs already reported to enhance effects of TMZ against glioma or glioma cell lines. Third, WST-8 and sphere-forming assays were performed using the two GSC lines (KGS01, KGS03) treated at lower concentrations (0.1, 0.5, and 1 µM) of the selected compounds with or without TMZ. Candidate drugs were selected based on demonstration of the viable cell rate reduction by enhancing the effect of TMZ even at the lowest concentration (0.1 μM). The concentration of TMZ used was 50 μM, which was selected based on TMZ blood concentration in clinical use (150 mg/m^2^–200 mg/m^2^; 43.3 μM–78.8 μM).

### Cell viability assay (WST-8 assay)

Cell viability of GSCs was assessed using Cell Counting Kit-8 (CCK-8; Dojindo, Kumamoto, Japan) following the manufacturer’s protocols. Spheres of GSCs were dissociated into single cells with StemPro Accutase (Gibco) and gently pipetting. For the first screening, 1.5 × 10^3^ cells were seeded with 36 μL culture medium in each well of a 384-well plate. At the second steps of screening, 2 × 10^3^ cells were seeded with 200 μL culture medium in each well of a 96-well Costar ultra-low attachment plate (Corning). After 48 h of treatment, the reagent of CCK-8 (10% amount of culture medium) was added and measured absorbance with a microplate reader to calculate the relative numbers of viable cells.

For GBM cell lines, we performed an Alamar blue assay (Biosource, Camarillo, CA, USA) in accordance with the manufacturer’s instructions. 1 × 10^3^ GBM cells of each cell line with 200 μL of culture medium supplemented with 0.1% FBS were seeded into each well of a 96-well plate. 20 μL (10% amount of culture medium) of Alamar blue was added to each well after 4 h of incubation at 37 °C with 5% CO_2_. The plate was read on a fluorescence plate reader at five timing (0, 24, 48, 72, and 96 h). The average fluorescence values from eight wells at each concentration were calculated and plotted in both assays. To estimate the TMZ-enhancing effect of kenpaullone on cell viability, cells were treated with kenpaullone (Wako, Osaka, Japan) alone, or in combination with TMZ.

### Western blotting analysis

Following treatment of cells with the indicated reagent(s), protein samples were extracted using ice-cold lysis buffer (Sigma-Aldrich) containing protease inhibitors and phosphatase inhibitors (Sigma-Aldrich). An aliquot containing 15 µg of extracted protein was analyzed by western blotting for expression level of proteins of interest with the corresponding specific antibodies (Supplementary Table S1), as previously described^48^.

### Tumor sphere-forming assay

We performed sphere-forming assay with same procedures as described previously^49–51^. In brief, we dissociated spheres of GSCs into single cells with StemPro Accutase at first. Then, we seeded 1 × 10^3^ single cells with 200 μL of medium for neurosphere mixed with 1.0% methylcellulose in each well of a 96-well Costar ultra-low attachment plate. GSCs were treated with kenpaullone alone or in combination with TMZ. Dimethyl sulfoxide (DMSO) was used as a control. After 10 days of incubation, sphere diameter was measured using a BZ-X Analyzer (KEYENCE) and sphere formation was estimated by scoring the number of spheres larger than 150 μm.

### Apoptosis assay

Apoptosis assay was performed using the Annexin V-FITC detection kit according to the manufacture’s protocol (abcam, Cambridge, UK). Glioma cells were seeded at a density of 1 × 10^5^ cells/2 mL/well onto a 6-well plate with culture medium containing kenpaullone with or without TMZ, or DMSO as a control. The cells were treated for 48 h and Annexin V-FITC positive cells were measured by flow cytometry (Gallios; Beckman Coulter Inc., Brea, CA, USA) to determine apoptosis.

### Mouse model of GBM and treatment

Animal experiments were performed in accordance with the same protocol as our previous study approved by the institutional review board^52^. To generate tumors, we transplanted 5 × 10^4^ KGS01 cells into brains of nude mice (BALB/cSlc-nu/nu, Charles River Laboratories, Osaka, Japan). Briefly, we anesthetized using pentobarbital (60 mg/kg), and pierced a burr hole into the skull 3 mm lateral from bregma with a micro drill, then injected cells stereotaxically at a depth of 3 mm below the dura mater. Mice were randomly assigned to four groups (n = 4 for each group) and treated with one of the following, kenpaullone, TMZ, combination of TMZ and kenpaullone, and DMSO as a control group. Treatment started from two days after transplantation and all mice were treated on the first 5 days of every month. Drugs were dissolved in 200 µL of 5% DMSO diluted by phosphate buffered saline (PBS) at 0.33 µg/body for kenpaullone or 48.5 μg/body for TMZ and injected by intraperitoneally. The dose of drugs was determined corresponding to the concentration used *in vitro*. Assuming that body fluid accounts for 60% of body weight in each mouse^53^, the dose of 0.33 µg/body corresponds approximately to a concentration of 0.1 µM for kenpaullone and a total dose of 48.5 μg/body corresponds to a concentration of 25 μM TMZ. To clarify the difference between monotherapy and combination therapy at the point of euthanasia, TMZ and kenpaullone concentrations were minimized to better evaluate the effects of each *in vivo*. After 9 weeks of treatment, all 16 mice were euthanized.

Brain tissues were dissected and postfixed in 4% PFA overnight at 4 °C. Specimens were cut into coronal sections around the insertion point and embedded in paraffin to make 4-µm serial sections. The histologically comparable positions, the slides where the tumor showed largest area, were used for immunostaining. We measured the area of each tumor in sections with the greatest tumor area immunostained for nestin to compare the size of tumors in each group. To measure the area, we acquired images by BZ-X700 microscope (Keyence, Osaka, Japan) and digitally processed using Keyence analysis software (Keyence). To estimate overall survival time, we injected tumor cells into additional mice and randomly assigned them to the same four groups as above (n = 5 for each group).

The project was approved by institute for experimental animals of Kanazawa University advanced research center (approval number: 163758, 163761), and all animal experiments were performed in accordance with the Guidelines for the Care and Use of Experimental Animals at Kanazawa University and other relevant guidelines.

### Immunohistochemistry

4-μm thick tissue sections were fixed on silanized slides. After deparaffinization, the specimens were put into citrate buffer (pH 6.0) and heated by autoclave at 121 °C for 10 min to activate antigen, treated with 0.3% hydrogen peroxide (H_2_O_2_) in methanol for 20 min to block endogenous peroxidase activity, and blocked for nonspecific binding for 1 h with 5% skim milk. The slides were reacted with each primary antibody (Supplementary Table S1) overnight at 4 °C, then washed with 1 × Tris Buffered Saline with Tween 20 (TBS-T), and immunostained using an Envision + kit (Dako, Tokyo, Japan) for 60 min at room temperature. Finally, the slides were color coded by using 3,3′-diaminobenzidine tetrahydrochloride (DAB) for 5 min, and sections were counterstained with hematoxylin.

### Statistical analysis

Statistical analyses were performed using Student’s t-test or Mann–Whitney U-test appropriately. The graph data are presented in the mean values and error bar representing standard deviation. Statistical significance of Kaplan-Meier survival curves was assessed using Log-rank analysis. Statistically significant differences were indicated by a *p* value ≤ 0.05. JMP software, version 10.0 (SAS institute Inc., Cary, NC, USA) was used for all statistical analyses described above.

### Ethics approval and consent to participate

Approval: All experimental protocols including animal experiments were approved by human genome/genetic analysis research ethics committee (Approval Number: 209), medical ethics committee (Approval Number: 2080, 2188), and institute for experimental animals of Kanazawa University (Approval Number: 163758, 163761). Accordance: All methods including animal experiments were carried out in accordance with relevant guidelines and regulations. Informed consent: Written informed consent was obtained from all participants or their representative related to this research.

## Supplementary information

Supplementary Information
