# Supplementary material for: Identification of GSK3β inhibitor kenpaullone as a temozolomide enhancer against glioblastoma
Source: Sci Rep. 2019 Jul 11;9:10049. doi: 10.1038/s41598-019-46454-8 (PMC6624278; doi:10.1038/s41598-019-46454-8)
Supplement: Supplementary file 1 — Supplementary Information [file 41598_2019_46454_MOESM1_ESM.pdf]

Identification of GSK3β inhibitor kenpaullone as a temozolomide enhancer against glioblastoma

Tomohiro Kitabayashi[1], Yu Dong[1,5], Takuya Furuta[2], Hemragul Sabit[1], Shabierjiang Jiapaer[1], Zhang Jiakang[1,5], Zhang Guangtao[1,6], Yasuhiko Hayashi[1], Masahiko Kobayashi[3], Takahiro Domoto[4], Toshinari Minamoto[4], Atsushi Hirao[3], and Mitsutoshi Nakada[1*]

[1] Department of Neurosurgery, Faculty of Medicine, Institute of Medical, Pharmaceutical and Health Sciences, Kanazawa University, Kanazawa, Japan

[2]Department of Pathology, Kurume University School of Medicine, Kurume, Japan

[3]Division of Molecular Genetics, Cancer Research Institute, Kanazawa University, Kanazawa, Japan

[4]Division of Translational and Clinical Oncology, Cancer Research Institute, Kanazawa University, Kanazawa, Japan

[5]Department of Neurosurgery, The First Affiliated Hospital of Harbin Medical University, Harbin, People's Republic of China

[6]Department of Neurosurgery, The First Hospital of Jilin University, Changchun, People's Republic of China

*** Corresponding author:**

Mitsutoshi Nakada

Supplementary Table 1. Source and working dilutions of the primary antibodies used in Western blotting, immunohistochemical and immunofluorescence staining

Antibody	MW (kDa)	Source	Working dilution	Company
β-actin	42	Mouse	1:5000 (WB)	WAKO
CD44	29-37	Mouse	1:400 (IF)	DAKO
CD133	97	Mouse	1:400 (IF)	R&D system
CDK1	34	Mouse	1:1000 (WB)	Cell Signaling
CDK2	33	Rabbit	1:1000 (WB)	Cell Signaling
c-Myc	49-57	Rabbit	1:5000 (WB)	abcam
DNMT3A	102-120	Mouse	1:500 (WB)	NOVUS
GFAP	55	Rabbit	1:600 (IF)	DAKO
GSK3β	46	Mouse	1:1000 (WB)	BD Biosciences
pGSK3β^{Y216}	46	Mouse	1:1000 (WB)	BD Biosciences
pGSK3β^{Y216}	47	Rabbit	1:2000 (IHC)	abcam
KLF4	65	Rabbit	1:1000 (WB)	Cell Signaling
Nestin	260	Mouse	1:400 (IHC)	BD Biosciences
Olig2	32	Rabbit	1:500 (IF)	IBL
PARP	116	Rabbit	1:1000 (WB)	Cell Signaling
cleaved PARP	89	Rabbit	1:1000 (WB)	Cell Signaling
SOX2	34	Rabbit	1:2000 (WB),	Gene Tex
Tuj1	55	Mouse	1:200 (IF)	R&D system

Abbreviations: CDK1/2; cyclin-dependent kinase 1/2, DNMT3A; DNA methyltransferase 3A, GFAP; glial fibrillary acidic protein, GSK3β; glycogen synthase kinase 3β, pGSK3β^{Y216}; GSK3β phosphorylated in tyrosine (Y) 216 residue, WB; Western blotting, IF; immunofluorescence, IHC; immunohistochemistry, KLF4; Kruppel-like factor 4, PARP; poly ADP-ribose polymerase, SOX2; SRY (sex determining region Y)-box 2

Supplementary Figure 1. Stemness, differentiation, and tumor-initiating ability of KGS03.

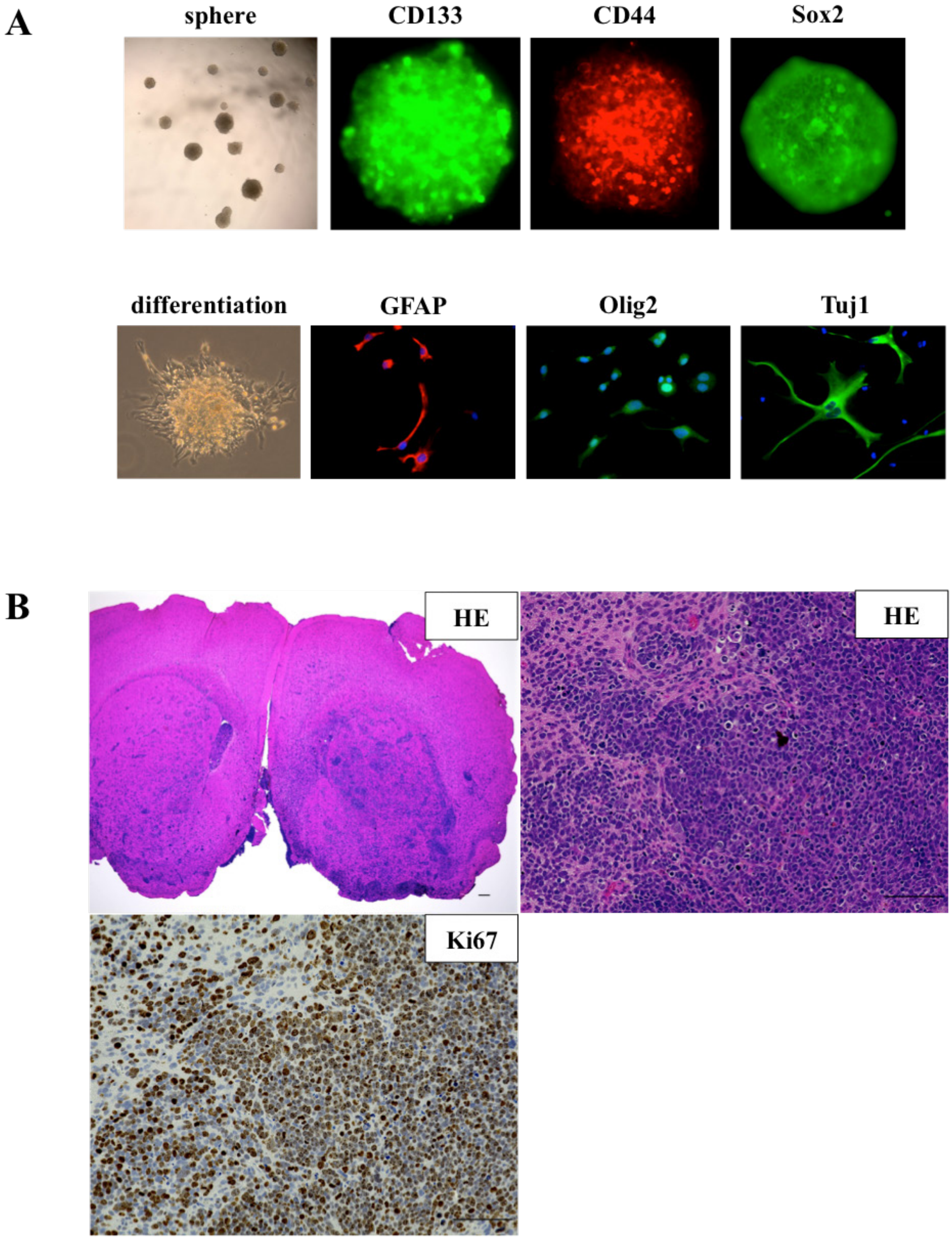

A, Tumor spheres derived from a GBM surgical specimen (KGS03) were positively stained for CD133, CD44, and SOX2, which are the characteristic markers of mesenchymal-type stem cells (upper panels). Magnification, ×400. Tumor spheres differentiated into GFAP- and Olig2-positive astrocyte-like cells and Tuj1-positive neuron-like cells in DMEM supplemented with 10% FBS (lower panels). Magnification, ×200. **B,** KGS03 cells (5×10^4) were transplanted into the mouse brain. A brain tumor developed 60 days following transplantation and histologically recapitulated the features of the original GBM. *Scale bar* = 100 μm.

Supplementary Figure 2. Western blotting analysis to investigate the effects of kenpaullone on GSK3β and CDKs in GBM cell lines.

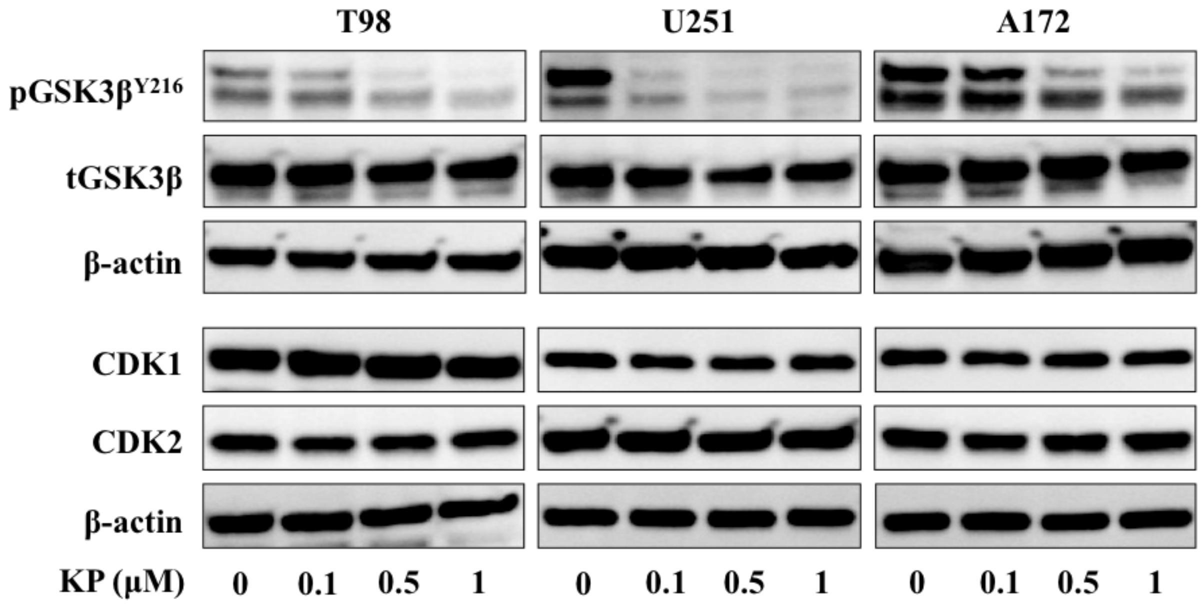

GBM (T98, U251, and A172) cells were treated with either DMSO (0) or kenpaullone (KP) at the indicated concentrations for 24 h. Phosphorylation and expression of GSK3β and expression of CDK1 and CDK2 in the respective cells were examined by Western blotting. Expression of β-actin was monitored as a loading control. Bands were cropped from different parts of the same gel, or from different gels.
